# *Cryptococcus neoformans* Infection Induces IL-17 Production by Promoting STAT3 Phosphorylation in CD4^+^ T Cells

**DOI:** 10.3389/fimmu.2022.872286

**Published:** 2022-05-27

**Authors:** Xiaoman Guo, Xinru Mao, Di Tian, Yixin Liao, Bintao Su, Chaoliang Ye, Dongling Shi, Tie Fu Liu, Yun Ling, Yi Hao

**Affiliations:** ^1^Department of Pathogen Biology, School of Basic Medicine, Tongji Medical College, Huazhong University of Science and Technology, Wuhan, China; ^2^Scientific Research Center, Shanghai Public Health Clinical Center, Fudan University, Shanghai, China; ^3^Department of Laboratory Medicine, Wuhan No. 1 Hospital, Tongji Medical College, Huazhong University of Science and Technology, Wuhan, China; ^4^Department of Infectious Disease, Shanghai Public Health Clinical Center, Fudan University, Shanghai, China; ^5^Department of Geriatrics, Tongji Hospital, Tongji Medical College, Huazhong University of Science and Technology, Wuhan, China

**Keywords:** IL-17, STAT3, CD4^+^ T cells, *Cryptococcus neoformans* meningitis, phosphorylation

## Abstract

*Cryptococcus neoformans* infection in the central nervous system is a severe infectious disease with poor outcomes and high mortality. It has been estimated that there are 220,000 new cases each year. Over 90% of *C. neoformans* meningitis cases were diagnosed in AIDS patients with CD4^+^ T cell count <100 cells/μl; however, the mechanism of cryptococcal meningitis in patients with normal immune functions remains unclear. IL-17 is a pro-inflammatory cytokine and plays an important role in anti-fungal immunity. Here we report that significantly high levels of IL-17 were predominantly detected in the cerebrospinal fluid of patients with either AIDS- or non-AIDS-associated *C. neoformans* meningitis but not in patients with tuberculous meningitis or non-neurosyphilis. Antifungal therapy minimized the IL-17 level in the cerebrospinal fluid. An *in vitro* mechanistic study showed that *C. neoformans* stimulation of healthy peripheral blood mononuclear cells prompted IL-17 production, and CD4^+^ T cells were the predominant IL-17-producing cells. IL-17 production by *C. neoformans* stimulation was STAT3 signaling dependent. Inhibition of STAT3 phosphorylation attenuated the *C. neoformans*-mediated IL-17 expression. Our data highlighted the significance of CD4^+^ T cells in antifungal immunity and suggested IL-17 as a diagnostic biomarker of *C. neoformans* infection and STAT3 as a checkpoint for antifungal targeted therapies.

## Introduction

*Cryptococcus neoformans* (*C. neoformans*) meningitis (CM) is a systemic and opportunistic fungal infectious disease with morbidity and mortality between 10% and 25% in medically advanced countries (1) and is often diagnosed in immunocompromised patients relevant or irrelevant to HIV/AIDS (2). It contributes about 15% of AIDS-associated opportunistic infection (3). In recent years, more and more cases of non-AIDS-associated *C. neoformans* meningitis have been reported, and about 220,000 new cases and 181,000 deaths have been estimated each year (3).

Previous studies of anti-fungal immunity in animal models suggested that Th17-type response is important for survival in *C. neoformans* infection (4). Pulmonary infection with *C. neoformans* strain H99c increased the pulmonary IL-17 (commonly known as IL-17A) production (5), and the IL-17 level of cerebrospinal fluids (CSF) in HIV-1 infection-associated CM is significantly higher than that in tuberculous meningitis (TBM) (6). IL-17 is a cytokine of CD4^+^ T helper subset, T helper 17 (Th17) cells, and has originally received attention for its pro-inflammatory function in autoimmunity (7–9). Previous research in a mouse model of *C. neoformans* infection suggested that a Th17-type response and IL-17 production are important for modulating survival against cryptococcosis (4). Besides this, the genetic defects of the IL-17 signaling pathway contribute to severe mucocutaneous *Candida*, oropharyngeal *Candida*, and *Aspergillus* infections in humans (10–12).

The differentiation of Th17 cells is regulated by a variety of signal pathways, among which the JAK2/STAT3 signal pathway plays a key role. The activated CD4^+^ T cells produce IL-6 to stimulate the JAK2/STAT3 pathway (13), thereby inducing the expression of the lineage-specific master regulator RORγt to promote the differentiation of Th17 cells (14, 15). Impaired Th17 cell differentiation is a consistent immune defect in STAT3 hyper-IgE syndromes, which may mediate protection against *C. neoformans* (16).

STAT3 is a potential transcription factor that transduces extracellular signals such as growth factors and cytokines through interaction with polypeptide receptors on the cell surface (17). It is post-translationally activated mainly by tyrosine phosphorylation to form STAT3 dimer, translocate from the cytoplasm into the nucleus, and bind to sequence-specific DNA elements of target genes (18). STAT3 is a consistently expressed protein, but its production is rapidly increased through self-regulation upon activation, as its promoter contains a binding site for its own dimers (19, 20). However, the mechanism of IL-17 induction remains unclear in non-HIV-associated *C. neoformans* meningitis.

The aim of this study was to investigate the mechanism of IL-17 production and the clinical effect of anti-fungal therapy on it in human *C. neoformans* meningitis. We found that the CSF of *C. neoformans* meningitis contains a high level of IL-17, and anti-fungal treatment minimizes the CSF IL-17 level and improves the clinical outcomes. A mechanistical study showed that STAT3 phosphorylation is essential for IL-17 induction. Our findings suggest that IL-17 is an important component in anti-fungal immunity and a potential biomarker for the diagnosis of *C. neoformans* meningitis.

## Materials and Methods

### Study Design and Patients

This study was approved by the ethics committee of Shanghai Public Health Clinical Center, and informed consent was obtained from the participants. The patients were hospitalized at the Department of Infectious Disease in Shanghai Public Health Clinical Center. Healthy individuals were recruited from employee volunteers in Scientific Research Center of Shanghai Public Health Clinical Center. The CSF and blood samples and the clinical data were preserved in accordance with the Declaration of Helsinki and local legislations.

Eleven patients with non-AIDS-associated (HIV^-^CM) and five patients with AIDS-associated (HIV^+^CM) *C. neoformans* meningitis and fifteen patients with TBM (drug-resistant TB) participated in this study. Nine patients with non-neurotic syphilis served as the meningitis-free disease control. The above-mentioned diseases were diagnosed based on the typical clinical presentations of infection, together with neuroimaging characteristics and positive laboratory findings, including abnormal routine CSF biochemical examinations for CM and TBM, and positive specific antibodies or pathogen tests, according to the guidelines from the Centers for Disease Control and Prevention (CDC) and World Health Organization (WHO). The patients with pregnancy and secondary or primary immunodeficiency diseases were excluded. Only adult (>16 years) patients with confirmed infectious diseases were recruited in this study. Although the entire treatment will last around 1 year, in the current study, the patients with non-AIDS CM were under treatment for approximately 90 days. In the meantime, the patients with TBM were under medical treatment for about 100 days. The CSF and blood samples were collected on admission and at about 3 months after the pathogen-specific therapies. The laboratory findings for the participants were summarized in **Table 1**.

**Table 1:** Clinical laboratory indexes in patients and their changes after therapies.

Characteristics	HIV^-^CM (n=11)		HIV+CM (n=5)		TBM (n=15)		Syphilis (n=9)	reference range
	before/after	p value^a^		p value^b^	before/after	p value^c^		
**CSF test**								
Protein (mg/L)	1336.35±1370.66/625.94±619.79	0.02	932.08±612.06	0.63	1380.02±657.59/592.88±423.09	0.01	271.90±143.57	150.00-450.00
Chloride (mmol/L)	121.96±6.78/124.44±3.65	0.06	121.80±2.68	0.99	115.73±6.44/122.64±5.39	0.01	124.28±2.23	120.00-132.00
Glucose (mmol/L)	2.56±1.59/3.92±1.25	0.41	2.46±0.60	0.84	2.10±1.11/2.69±1.01	0.46	3.47±0.73	2.20-4.40
WBC (10^6^/L)	105.00±94.19/8.2±8.39	0.01	8.06±9.23	0.05	170.17±136.21/21.92±17.23	<0.0001	3.00±2.82	0-8x10^6^
RBC (10^6^/L)	18.10±30.93/47.20±121.28	0.01	3.00±4.24	0.30	119.75±264.98/294.80±908.44	0.01	6.38±21.99	0-10^6^
CSF pressure (mmH2O)	210.80±112.39/204.80±103.16	0.75	216.00±108.59	0.83	175.42±92.17/154.17±54.85	0.03	166.88±42.21	100.00-180.00
Cryptococcal Antigen Test	1:2560/neg		pos(1:2560)		neg/neg		neg	neg
**Blood Test**								
WBC (×10^9^/L)	10.53±7.14/7.71±2.99	0.02	2.50±0.76	0.02	7.43±2.17/6.47±2.54	0.39	7.44±3.20	3.50-9.50
Lymphocytes (×10^9^/L)	0.81±0.52/1.11±0.63	0.52	0.66±0.31	0.50	0.95±0.71/1.41±0.69	0.80	1.33±0.25	1.10-3.20
Monocytes (×10^9^/L)	0.52±0.31/0.50±0.24	0.47	0.28±0.04	0.14	0.31±0.16/0.47±0.22	0.24	0.37±0.23	0.10-0.60
RBC (×10^12^/L)	3.80±0.65/3.73±0.74	0.67	3.77±1.04	0.90	4.12±0.86/4.07±0.77	0.48	4.21±0.52	4.30-5.80
Platelet (×10^9^/L)	173.70±91.21/166.20±74.98	0.57	171.00±74.17	0.95	223.42±82.89/ 221.17±91.49	0.66	200.63±76.05	125.00-350.00
Basophils (×10^9^/L)	0.03±0.03/0.03±0.01	0.34	0.02±0.02	0.31	0.01±0.25/0.02±0.02	0.02	0.01±0.01	0-0.06
Eosinophils (×10^9^/L)	0.03±0.04/0.04±0.03	0.50	0.08±0.13	0.18	0.03±1.85/0.07±0.12	0.0007	0.04±0.06	0.02-0.52
Neutrophils (×10^9^/L)	9.26±7.13/6.35±3.75	0.05	1.62±0.62	0.03	6.14±2.04/4.50±2.33	0.38	5.69±3.26	1.80-6.30
CD3^+^ cell (cell/μl)	519.90±291.49/848.00±480.64	0.15	411.80±298.26	0.53	753.92±505.77/995.50±558.15	0.98	1032.00±532.69	690.00-2540.00
CD4^+^cell (cell/μl)	250.80±168.57/374.14±250.38	0.24	23.00±22.85	0.01	455.17±329.62/613.50±277.55	0.53	536.00±405.36	410.00-1590.00
CD8^+^cell (cell/μl)	240.80±165.09/434.71±238.14	0.28	355.40±251.74	0.30	243.00±149.03/426.30±172.31	0.02	426.83±256.05	190.00-1140.00
CD45^+^cell (cell/μl)	758.90±412.98/1117.00±610.24	0.25	598.00±380.38	0.46	1108.08±704.06/1435.60±609.06	0.33	1498.83±714.25	900.00-3500.00
C3 (g/L)	0.88±0.29/0.91±0.29	0.64	0.89±0.14	0.84	1.00±0.19/1.02±0.26	0.30	0.97±0.13	0.90-1.80
C4 (g/L)	0.23±0.07/0.20±0.07	0.83	0.21±0.09	0.63	0.24±0.07/0.22±0.09	0.43	0.21±0.08	0.10-0.40
IgA (g/L)	2.25±1.24/1.30±0.64	0.19	6.76±5.50	0.02	2.62±1.15/2.00±0.81	0.15	2.69±0.99	0.70-4.00
IgM (g/L)	1.79±2.48/0.44±0.29	0.01	2.08±2.24	0.79	1.04 ±0.49/0.96±0.35	0.36	1.76±0.72	0.40-2.30
IgG (g/L)	10.10±5.84/6.84±2.52	0.09	18.85±8.04	0.04	11.22±4.29/11.29±4.32	0.87	12.45±4.03	7.00-16.00
CRP (mg/L)	8.01±4.65/3.99±1.18	0.03	3.52±1.03	0.12	15.01±21.77/13.07±15.30	0.39	8.84±13.08	<3.00
Procalcitonin (ng/ml)	0.08±0.25/0.3±2.22	<0.0001	0.06±0.05	0.26	0.07±0.05/0.13±0.22	0.002	0.10±0.09	0-0.05
								
HIV antigens/antibodies	neg		pos		neg		neg	

Data are shown as mean ± SD.

CM, Cryptococcus neoformans meningitis; TBM, tuberculous meningitis; before, before therapy; after, after therapy; CSF, cerebrospinal fluid; WBC, white blood cells; RBC, red blood cells; CRP, C-reactive protein.

a P-value indicates HIV^-^CM before compared with HIV^-^CM after.

b P-value indicates HIV^-^CM before compared with HIV^+^CM.

c P-value indicates TBM before compared with TBM after.

### Preparation and Stimulation of Peripheral Blood Mononuclear Cell

Whole blood— anti-coagulated with heparin—from healthy individuals was diluted in 1:2 with RPMI 1640 and was centrifugated on Hypaque-Ficoll gradients (GE, density 1.077 g/ml) at 400*g* for 30 min. Peripheral blood mononuclear cells (PBMCs) were then collected and washed twice with phosphate-buffered saline (PBS). The cells were cultured in RPMI 1640 (Biological Industries) supplemented with 10% fetal bovine serum (Biological Industries) and 1% penicillin/streptomycin (Beyotime). PBMCs (1 × 10^6^/ml) were stimulated for the indicated times with or without *C. neoformans* (1 × 10^6^/ml, provided by our clinical laboratory) and bacillus Calmette–Guérin (BCG, 1 × 10^5^/ml, provided by Professor Feifei Wang, Fudan University). In specific experiments, PBMCs were pretreated for 30 min with 2.5 μM of STAT3 inhibitor Stattic (Abcam) before stimulation for phospho-STAT3 analysis by flow cytometry.

### Assessment of Th1, Th2, and Th17 Cytokines Using the Cytometric Bead Array

The quantitative evaluation of intracellular cytokines was performed using the Human Cytometric Bead Array (CBA) Th1/Th2/Th17 Cytokine Kit (BD Biosciences), according to the manufacturer’s protocol. Equal volumes of assay beads, detection reagent, and the studied sample or standard were added consecutively to each tube and incubated in the dark for 3 h at room temperature. The samples were then washed with 1 ml of wash buffer and centrifuged at 500*g* for 5 min. After discarding the supernatant, the pellet was resuspended in 300 µl of buffer and analyzed on the same day by flow cytometry. FCAP Array Software, version 3.0, from BD Biosciences was employed to translate the images into data.

### Flow Cytometry Analysis of Cell Surface Markers

PBMCs were incubated in FACS buffer (PBS supplemented with 0.5% bovine serum albumin) for 20 min at 4°C with the following antibodies: anti-CD4 (L200, BD Biosciences), anti-CD8 (RPA-T8, BD Biosciences), anti-CD3 (OKT3, Invitrogen), anti-γδTCR (B1.1, Invitrogen), and anti-CD19 (HIB19, eBioscience). The cells were subsequently washed twice and were resuspended with FACS buffer. The flow cytometry data was acquired on BD LSR Fortessa and analyzed by FlowJo V10. Dead cells were excluded by using Fixable Viability Dye (Invitrogen).

### Flow Cytometry Analysis of IL-17-Producing Cells

PBMCs (1 × 10^6^/well) were incubated in 24-well plates for 30 min or 3 days with different stimuli. Protein transport inhibitor cocktail (eBioscience) was added for 5 h before the end of the cell culture. After surface staining, the cells were fixed and permeabilized using an intracellular fixation and permeabilization buffer set (eBioscience). Intracellular IL-17 were stained with anti-IL-17 (IL-17A) antibodies (eBio64DEC17, eBioscience) according to the manufacturers’ protocols and analyzed by flow cytometry.

### Flow Cytometry Analysis of Intracellular Phospho-STAT3 and Phospho-STAT1

PBMCs were stimulated for 30 min with different stimuli. After surface marker staining, the cells were fixed with 4% paraformaldehyde (Biosharp) for 15 min at room temperature and permeabilized for a minimum of 10 min on ice by slowly adding ice-cold methanol (Sangon Biotech) to a final concentration of 90%. The cells were then stained with rabbit anti-human phospho-STAT3-Tyr_705_ monoclonal antibody (D3A7, Cell Signaling Technology) or human phospho-STAT1 (Tyr701) Rabbit mAb (58D6, Cell Signaling Technology) for 1 h and were followed by incubation with Alexa Fluor 488-goat anti-rabbit IgG (H+L) (Invitrogen) for 30 min at room temperature. The cells were then washed in PBS twice and were resuspended in 300 μl PBS supplemented with 0.5% bovine serum albumin for the flow cytometry analysis.

### Enzyme-Linked Immunosorbent Assay

The levels of IL-6, IL-10, IL-17, and IFN-γ in the CSF and serum of patients with meningitis, syphilis, and healthy controls, and the levels of IL-17 and IFN-γ in the cell culture supernatants were measured by enzyme-linked immunosorbent assay (ELISA) using ELISA kits of human IL-6 (Absin), human IL-10 (Absin), human IL-17 (Abcam), and human IFN-γ (Abcam), respectively. The OD values at 450 nm were read in duplicates using an automatic microplate reader (BioTek, Synergy2). Serial dilutions of recombinant cytokines were used to generate a standard curve.

### Western Blot

PBMCs were lysed on ice for 30 min using RIPA lysis buffer (Beyotime) with protease inhibitors (Beyotime) and phosphatase inhibitors cocktail (Beyotime), and the protein concentration was determined by a bicinchoninic acid assay kit (Biosharp). The proteins (50 µg per lane) were mixed with one-fifth volume of 5× loading buffer, separated by 10% SDS-PAGE, and were then transferred onto polyvinylidene difluoride membranes (Millipore). The membranes were blocked for 1 h with 5% nonfat milk at room temperature and were incubated overnight at 4°C with primary antibodies against STAT3 rabbit mAb (79D7, Cell Signaling Technology), phospho-STAT3-Tyr_705_ rabbit mAb (D3A7, Cell Signaling Technology), NF-κB p65 rabbit mAb (D14E12, Cell Signaling Technology), anti-NF-kB p65-phospho-S_536_ antibody (EP2294Y, Abcam), and β-actin rabbit monoclonal antibody (Beyotime). After 3 washes with Tris-buffered saline with 0.1% Tween® 20 detergent, the membranes were incubated for 1 h at room temperature with anti-rabbit horseradish peroxidase-conjugated secondary antibody (GE) and were detected with enhanced chemiluminescence (Beyotime). The results were analyzed using Image Studio software.

### Statistical Analyses

The statistical differences between the two groups were analyzed by Student’s *t*-test using GraphPad Prism Software 8.0 (GraphPad Software, Inc), and the statistical differences among multiple groups were analyzed by Tukey’s *post*-*hoc* test. *P*-value <0.05 was considered a statistically significant difference. Data were presented as mean ± standard deviation or standard error.

## Results

### Pathogen-Specific Therapies Restore Clinical Laboratory Abnormalities

The confirmed meningitis patients including 11 CM (7 male and 4 female patients) and 15 TBM (9 male and 6 female patients) had been treated according to the Infectious Diseases Society of America guidelines. The CM patients received a combined anti-fungal therapy of fluconazole, flucytosine, and amphotericin B (21), and the TBM patients were treated with first-line anti-tuberculous drugs of rifampin, isoniazid, ethambutol, and pyrazinamide. The first-line drug-resistant patients were subsequently treated with the second-line drugs moxifloxacin and cycloserine (22). Besides this, all meningitis patients were given a corticosteroid therapy including prednisolone and dexamethasone in the early stage of the disease to relieve acute inflammatory responses and brain edema. The CSF and blood samples were examined before and after therapies. The clinical laboratory characteristics were compared in **Table 1**. Anti-fungal therapy cleared the CSF pathogen and significantly decreased the levels of protein, white blood cell (WBC), and red blood cell (RBC) counts in the CSF, WBC and neutrophil counts, and IgM, CRP, and procalcitonin in peripheral blood. Similarly, the anti-tuberculous therapy significantly decreased the levels of protein, chloride, WBC, and RBC counts in the CSF and the cell counts of basophils, eosinophils, and CD8^+^ T cells and procalcitonin in peripheral blood. All laboratory results of meningitis-free syphilis cases were in the normal range and were not re-checked after the anti-syphilis therapy.

### *C. neoformans* Meningitis Selectively Increases CSF IL-17

**Figure 1:**
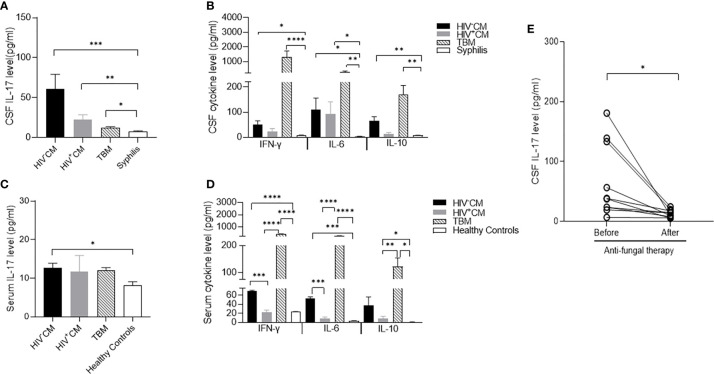
Cytokine levels of cerebrospinal fluid (CSF) and serum in patients with *C. neoformans* and *tuberculosis* meningitis. CSF was collected before and at about 3 months after the pathogen-specific therapies, while serum samples were collected on admission. The cytokine levels were measured by ELISA. **(A)** CSF levels of IL-17 in non-HIV CM (*n* = 11), HIV-positive CM (*n* = 5), TBM (*n* = 15), and syphilis (*n* = 9) on admission. **(B)** CSF levels of IFN-γ, IL-10, and IL-6 in non-HIV CM (*n* = 11), HIV-positive CM (*n* = 5), TBM (*n* = 15), and syphilis (*n* = 9) on admission. Serum levels of IL-17 **(C)** and IFN-γ, IL-10, and IL-6 **(D)** in healthy controls (*n* = 3), non-HIV CM (*n* = 4), HIV-positive CM (*n* = 5), and TBM (*n* = 4) at admission. **(E)** Changes of CSF levels of IL-17 in non-HIV CM (*n* = 11) and TBM (*n* = 15) before and after pathogen-specific therapies. Statistical significance was indicated as ^*^p < 0.05, **p < 0.01, ***p < 0.001, ^****^p < 0.0001. Data are shown as mean ± SE of the samples. CM, *C. neoformans* meningitis; TBM, tuberculous meningitis.

To determine the cytokine expression of meningitis, we compared the CSF levels of proinflammatory cytokines IL-6, IL-17, and IFN-γ and anti-inflammatory cytokine IL-10 in patients with non-HIV and HIV-positive CM, TBM, and patients with meningitis-free syphilis. In comparison with the trace CSF levels of all 4 cytokines in non-neurotic syphilis, both non-HIV and HIV-positive CM and TBM significantly increased the CSF IL-6 and IL-10 levels, and TBM considerably increased the CSF IFN-γ and slightly increased the IL-17 levels. In contrast, non-HIV and HIV-positive CM selectively increased the CSF IL-17 level with a minimal induction of IFN-γ (**Figures 1A, B**). Similar to the findings in the CSF, both non-HIV and HIV-positive CM and TBM significantly induced serum IL-6, IL-10, and IFN-γ. In contrast, non-HIV CM, but not TBM, significantly induced serum IL-17 in comparison with the healthy controls (**Figures 1C, D**).

We next followed the changes of CSF cytokine levels in response to pathogen-specific therapies. Consistent with the clinical improvement, the anti-fungal therapy minimized the CSF IL-17 level of non-HIV CM compared to that in meningitis-free syphilis (**Figure 1E**). The anti-fungal therapy also diminished CSF IL-6, IL-10, and IFN-γ (**Supplementary Figure S1A**). The anti-tuberculous therapy restored the CSF IFN-γ, IL-6, and IL-10 to basal levels but did not change the CSF IL-17 level (**Supplementary Figure S1B**).

### Cytokine Profiles of PBMCs in Response to *C. neoformans*

**Figure 2:**
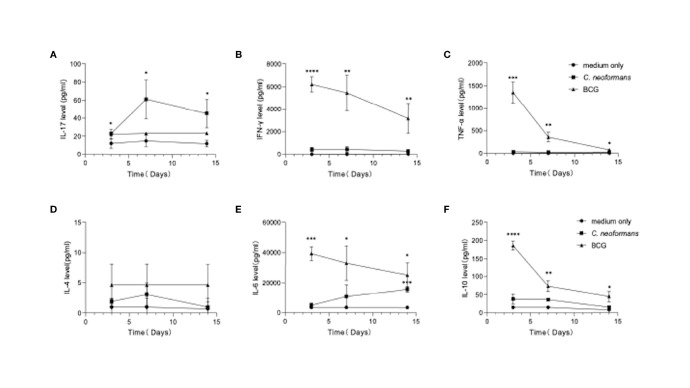
Dynamics of cytokine production by peripheral blood mononuclear cells (PBMCs) in response to *C. neoformans* or bacillus Calmette–Guérin (BCG) stimulation. Healthy PBMCs were stimulated with *C. neoformans* or BCG for different times (day 3/7/14). The supernatant cytokine levels were measured by ELISA. The dynamics of IL-17 **(A)**, IFN-γ **(B)**, TNF-α **(C)**, IL-4. **(D)**, IL-6 **(E)**, and IL-10 **(F)** are shown as mean ± SE of three individual experiments. *0.01<p<0.05, **0.001<p<0.01, ***<0.0001<p<0.001, ****p<0.0001

To model an *in vivo* infection, we next stimulated healthy PBMCs for 3, 7, or 14 days with *C. neoformans* or BCG, which, as an efficient vaccine, is well known to best mimic *in vivo* conditional pathogenic infections and stimulate effective anti-tuberculosis immunity. Here BCG served as a stimulus to induce an anti-bacterial response *in vitro*. The levels of Th1 (IFN-γ and TNF-α) (**Figures 2B, C**), Th2 (IL-4, IL-10, and IL-6) (**Figures 2D–F**), and Th17 (IL-17) (**Figure 2A**) cytokines were followed by flow cytometric bead array. *C. neoformans* stimulation significantly induced IL-17 at day 3, which peaked at day 7 and was maintained at a high level at day 14, but it did not significantly change the levels of other cytokines, except IL-6 that was significantly increased at day 14. In contrast, BCG stimulation significantly induced IFN-γ, TNF-α, and IL-6 and IL-10 at day 3 and gradually decreased their levels thereafter; however, BCG did not significantly induce IL-17 production. Thus, in contrast to the fast Th1/Th2 response by BCG stimulation, *C. neoformans* stimulation induces a postponed Th17 response.

### *C. neoformans* Stimulates IL-17 Production by CD4^+^ T Cells

**Figure 3:**
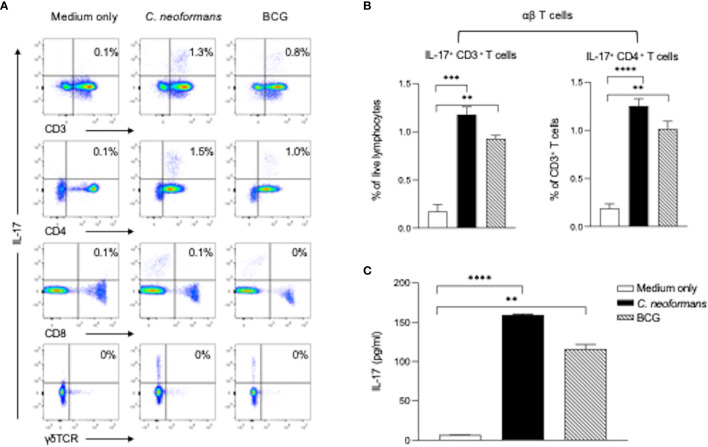
*C. neoformans* stimulates IL-17 production by CD4^+^ T cells *in vitro.* Peripheral blood mononuclear cells were stimulated with *C. neoformans* or bacillus Calmette–Guérin for 3 days with protein transport inhibitor cocktail for 5 h before the cells were collected. The IL-17-producing cells were analyzed by flow cytometry: representative data of IL-17^+^ T cells in αβTCR^+^ T cells (CD3^+^γδTCR^-^ cells) and γδTCR^+^ T cells **(A)** and percentages of IL-17^+^ CD3^+^ cells in live lymphocytes and percentages of IL-17^+^ CD4^+^ cells in live CD3^+^ cells **(B)**. The corresponding IL-17 level in the supernatant **(C)** is shown as mean ± SE of three individual experiments. ^**^p < 0.01, ^***^p < 0.001, ^****^p < 0.0001.

To determine cytokine-producing cells, we next stimulated healthy PBMCs for 3 days with *C. neoformans* or BCG and analyzed IL-17-producing cells in αβT (CD4^+^ or CD8^+^ T cells) and γδT (CD3^+^γδTCR^+^) subpopulations by flow cytometry assay. *C. neoformans* stimulation significantly increased the IL-17-producing cells in the CD4^+^ T subset but not in the CD8^+^ or γδTCR^+^ subpopulations. In contrast, BCG marginally induced IL-17 positive cells (**Figures 3A, B**). To confirm the flow cytometry data, the supernatant IL-17 level in *C. neoformans* or BCG-stimulated cultures were compared by ELISA. As shown in **Figure 3C**, the IL-17 level was significantly induced by *C. neoformans* in contrast to the slight increase by BCG stimulation. Consistent with the CSF data, IFN-γ was considerably induced by BCG but minimally induced by *C. neoformans* stimulation (**Supplementary Figure S2**).

### *C. neoformans* Triggers STAT3 Phosphorylation

**Figure 4:**
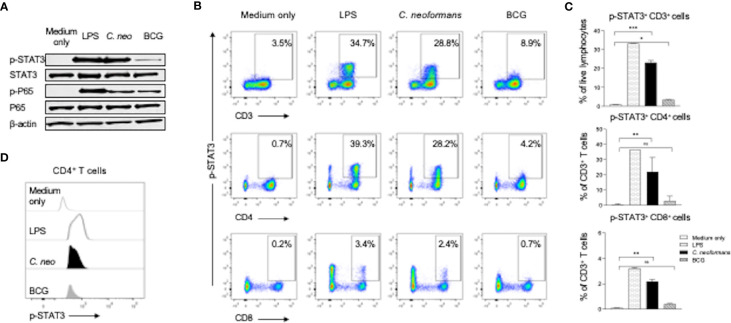
*C. neoformans* stimulates the phosphorylation of STAT3 in CD4^+^ T cells. Peripheral blood mononuclear cells were stimulated for 30 min with lipopolysaccharide (100 ng/ml), *C. neoformans*, or BCG. The phosphorylation status of STAT3 and its downstream P65 at the protein level were analyzed by western blot **(A)**. Phosphorylation of STAT3 in T cell subsets **(B, C)**, and its fluorescence intensity was analyzed by flow cytometry **(D)**. Representative data of three individual experiments are shown. *0.01<p<0.05, **0.001<p<0.01, ***<0.0001<p<0.001.

To investigate the mechanism of IL-17 production by *C. neoformans* stimulation, we stimulated healthy PBMCs for 30 min with *C. neoformans* or BCG and analyzed the changes of the phosphorylation status of STAT3 and its downstream p65 by western blot analysis. Lipopolysaccharide (LPS, 100 ng/ml) stimulation was set as the positive control. As shown in **Figure 4A**, both LPS and *C. neoformans* significantly stimulated the phosphorylation of STAT3, in contrast to the negligible phospho-STAT3 by BCG; however, both *C. neoformans* and BCG were equally effective in stimulating p65 phosphorylation, suggesting that they activate the p65-dependent pathway *via* different signaling (**Figure 4A**). To determine if *C. neoformans*-mediated STAT3 phosphorylation occurs in IL-17-producing cells, we analyzed the phospho-STAT3^+^ T cell subpopulations by flow cytometry analysis. We found that, among T cells, about 30% of CD4^+^ T subpopulation, but only about 3% of CD8^+^ T subpopulation, was phospho-STAT3-positive in LPS or *C. neoformans*-stimulated cultures, whereas BCG stimulated STAT3 phosphorylation only in about 4% of CD4^+^ T cells and 0.7% of CD8^+^ T cells (**Figures 4B, C**). The densitometry analysis showed that *C. neoformans* stimulated a significantly higher fluorescence intensity of phospho-STAT3 than BCG did in CD4^+^ T cells (**Figure 4D**). To further confirm whether the phosphorylation of STAT3 was specific for *C. neoformans* stimulation, we analyzed the phospho-STAT1^+^ T cell by flow cytometry (**Supplementary Figure S3**). The results showed that about 0.7% of CD4^+^ T cells were phospho-STAT1-positive in response to *C. neoformans* stimulation and in contrast to the nearly 3.2% of phospho-STAT1^+^CD4^+^ T cells under BCG stimulation (**Supplementary Figures S3A, B**), which was consistent with the results of the mean fluorescence intensity analysis (**Supplementary Figure S3C**). Moreover, to verify the relationship of STAT3 phosphorylation and IL-17 production at an early stage, we detected intracellular IL-17 expression in T cells. We found that, similar to STAT3 phosphorylation, IL-17 was produced especially by CD4^+^ T cells, but not by other cells, in response to *C. neoformans* (**Supplementary Figure S4**). Taken together, our data indicate that CD4^+^IL-17^+^ cells participate in cryptococcal immune response *via* STAT3 phosphorylation.

### Inhibition of STAT3 Phosphorylation Diminishes *C. neoformans*-Mediated IL-17^+^ CD4^+^ T Cells

**Figure 5:**
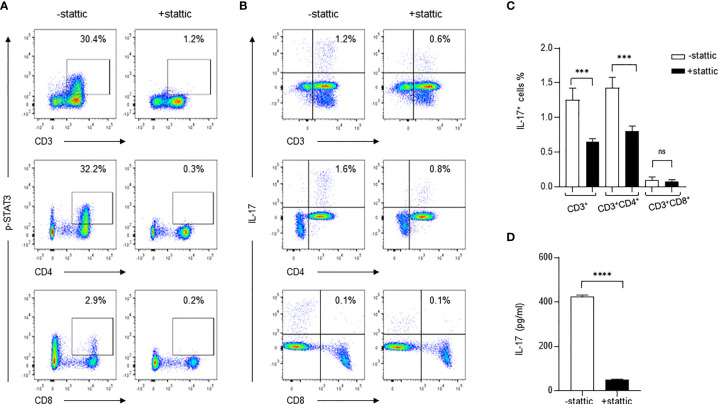
Inhibition of STAT3 phosphorylation diminishes IL-17 production in response to *C. neoformans*. Peripheral blood mononuclear cells were pretreated for 30 min with STAT3 phosphorylation inhibitor stattic (2.5μM) and were subjected to stimulation for 3 days with *C. neoformans*. Changes of the phosphorylated STAT3 **(A)** and IL-17-producing cells **(B, C)** were analyzed by flow cytometry assay, and the supernatant IL-17 level (mean ± SE) was evaluated by ELISA **(D)**. Representative data of three individual experiments are shown. ***0.0001<p<0.01, ^****^p < 0.0001; ns, no significance.

To determine if the STAT3-dependent pathway is exclusively important for IL-17 production during *C. neoformans* stimulation, we used a small molecule inhibitor stattic (2.5 μM) to specifically inhibit STAT3 phosphorylation and followed the changes of IL-17^+^ CD4^+^ T cells during *C. neoformans* stimulation of healthy PBMCs. As shown in **Figure 5A**, *C. neoformans* stimulation induced p-STAT3 in 30.4% of total T (CD3^+^) cells, 32.2% of CD4^+^ T cells, and 2.9% of CD8^+^ T cells; pretreatment of PBMCs for 30 mins with stattic, p-STAT3^+^CD3^+^, p-STAT3^+^CD4^+^, and p-STAT3^+^CD8^+^ cells resulted in dropping to 1.2, 0.3, and 0.2%, respectively. In accordance with the changes of STAT3 phosphorylation, *C. neoformans* stimulation yielded 1.2% IL-17^+^ CD3^+^ cells in live lymphocytes and 1.6% IL-17^+^ CD4^+^T in αβT cells, which were respectively reduced to 0.6 and 0.8% by stattic pretreatment (**Figures 5B, C**). To confirm the flow cytometry data, *C. neoformans* stimulation for 3 days produced over 400 pg/ml of supernatant IL-17 that was diminished by stattic pretreatment (**Figure 5D**). These observations collectively suggested that *C. neoformans* stimulates IL-17 production by CD4^+^ T cells *via* the STAT3-dependent signaling pathway.

## Discussion

The pathogenesis of fungal infections and anti-fungal immunity have recently attracted more and more attentions and effective anti-fungal therapies have become an urgent clinical need. There are more than 300 human pathogenic fungi, and immunodeficiency patients are more susceptible to fungal infections. With the increasing prevalence of HIV infection worldwide, opportunistic fungal infection has been the main driver of HIV mortality. In Africa, *C. neoformans* meningitis accounts for about 70% (23) of the global deaths of AIDS-associated fungal infections, including candidiasis, *Penicillium marneffei* infection, pulmonary aspergillus, and so on (24). In recent years, more and more non-HIV-infected sufferers have been reported with CM (25, 26).

IL-17 production has been proposed as an important mechanism of anti-fungal immunity. This inflammatory cytokine IL-17 could be secreted by CD4^+^, CD8^+^, γδ T, and NK cells (27, 28). In a mice model of *C. neoformans* infection, CD4^+^ T cells were a crucial component of cell-mediated fungal clearance (29–31). In humans, majority of *C. neoformans* infections were related with CD4^+^ T cell deficiency due to HIV co-infection, while IL-17-producing CD4^+^ T (Th17) cells were needed for vaccine-mediated protection against *C. neoformans* (32–34). Consistent with previous findings, our research suggested that IL-17 was secreted especially by CD4^+^ T cells, but not by other cells, in response to the stimulation of *C. neoformans*. Although the CD4^+^ cell counts in HIV-negative CM patients were lower than the normal reference range but much higher than those of HIV-positive CM patients, all non-AIDS-associated CM patients in the current study had no evidence of immunodeficiency. In addition, previous studies had found that *in vitro* Th1-type cytokine IFN-γ also plays a significant role to enhance the phagocytic activity of macrophages against *C. neoformans* (35–37). A mouse model of *C. neoformans* H99 gamma strain infection produced IFN-γ that protected the invasion of *C. neoformans* pulmonale (5, 38).

Predominantly increased CSF IL-17 has been reported in patients with HIV^+^ *C. neoformans* meningitis (6, 39). Consistent with those observations, our data showed that the CSF IL-17 level was selectively increased in CM patients with or without HIV, but not in TBM patients whose CSF contained a high level of IFN-γ. Interestingly, the CSF IL-17 in non-HIV CM patients was significantly elevated compared with that of HIV-positive CM, which indicated preferential Th17 responses to *C. neoformans* in immunocompetent individuals and led to speculations of other sources of IL-17-producing cells in case of CD4^+^ T cell deficiency. We have also found a slightly higher-than-normal serum IL-17 level in non-HIV CM patients, but not in HIV-positive CM. The increased CSF IL-17 or IFN-γ in *C. neoformans* or tuberculous meningitis was minimized by anti-fungal or anti-tuberculous therapies, respectively. However, the mechanism of IL-17 in the pathogenesis of *C. neoformans* meningitis in non-AIDS patients is not clear.

It is well known that the pro-inflammatory cytokine production by infections involves cell interactions between immune cells (40–42). To study the immune function of human fungal infection, we used fungus-stimulated healthy PBMC to mimic the *in vivo* situation and investigated the dynamics of IL-17 production and its signaling pathway. We found that *C. neoformans* stimulation induced high levels of IL-17 both in the cell and the supernatant, and T cells, especially CD4^+^ T cells, were the only IL-17 producer; no IL-17^+^CD8^+^ T cells or IL-17^+^γδ T cells were detected.

Protein phosphorylation is one of the post-translational modifications that are particularly important for the regulation of cellular activities (43). NF-κB P65 is a well-known gene transactivator in the innate immune signaling pathway against infection (44). We found that it participates in anti-fungal immunity by showing its phosphorylation activation by cryptococcal stimulation. An earlier study had linked the STAT3 signaling pathway with differentiation of naive CD4^+^ T cells to Th17 phenotype and IL-17 production (45–47). We demonstrated here that STAT3 signaling plays an important role in the development of IL-17-secreting cells in response to fungal stimulation. In contrast to trace phospho-STAT3 in CD4^+^ T cells by BCG stimulation, *C. neoformans* stimulation selectively induced the phosphorylation of STAT3 mainly in CD4^+^ T cells. No phospho-STAT3-positive cells were detected in CD8^+^ or γδ T cell subsets. Previous studies suggested that STAT1 is mainly involved in IFN-γ induction. This is aligned with our findings that phospho-STAT1 in T cells is not associated with *C. neoformans* stimulation (48, 49). However, BCG is a live attenuated tuberculosis vaccine. Although it can produce effective anti-tuberculosis immunity and has well reproduced the immune response of TBM in the current *in vitro* PBMC stimulation, it has a defect in stimulating T cell immunity due to the deletion of germline DNA fragments called region of difference (RD)-1, -2, -3, and -4, which encode virulent genes in the wild-type tuberculosis strain Mtb H37Rv (50, 51). In particular, RD-1 encodes two secretory proteins, CFP-10 and ESAT-6 (52–54), both of which have T-cell activation and macrophage deactivation functions (55). Thus, the difference of full immune response by infections of BCG and Mtb H37Rv strains should be carefully evaluated in the future.

The STAT3-dependent IL-17 production was further confirmed by the pretreatment of cells with a small molecule compound stattic, a selective STAT3 inhibitor that inhibits the activation, dimerization, and nuclear translocation of STAT3 by interacting with its SH2 domain (56, 57) and represses STAT3 phosphorylation (58). In the current study, the pretreatment of PBMCs significantly repressed STAT3 phosphorylation at Tyr705 by cryptococcal stimulation in CD4^+^ T cells and further diminished the IL-17-producing CD4^+^ T cells and secretory IL-17 in the cell culture supernatant. In addition, *C. neoformans* stimulation selectively triggers STAT3 phosphorylation rather than STAT1 phosphorylation, which further confirmed the STAT3-dependent IL-17 production.

Signal transduction to gene transcription to protein translation is a complex regulatory process. It is common in all signaling pathways that the phosphorylation of upstream signal molecules occurs almost immediately after the activation of the signaling pathway. Thus, as the very upstream of IL-17-producing signaling pathway, STAT3 was rapidly phosphorylated in response to *C. neoformans* stimulation, whereas IL-17 protein expression was significantly postponed probably due to the down-stream multiple regulatory processes, such as the transcription of target gene and/or daughter genes to finally induce IL-17 gene transcription and translation (59).

In conclusion, our study demonstrated that *C. neoformans* infection stimulates the development of Th17 cells to produce IL-17 by activating the STAT3-dependent signal pathway, and IL-17 could be a potential biomarker of and STAT3 a checkpoint of targeted therapies for fungal infection.

## Data Availability Statement

The raw data supporting the conclusions of this article will be made available by the authors without undue reservation.

## Ethics Statement

The studies involving human participants were reviewed and approved by the Ethical Committee of Shanghai Public Health Clinical Center [approval number (2020) 2020-S144-01]. The patients/participants provided their written informed consent to participate in this study.

## Author Contributions

YH and YLing conceptualized this study and designed the experiments. XG performed the experiments and data analysis. XM helped in the flow cytometry assays. DT, YL, BS, CY, and DS collected the CSF and blood samples. XG, TL, YLing, and YH wrote the manuscript. All authors contributed to the article and approved the submitted version.

## Funding

This work was sponsored by the National Natural Science Foundation of China (82072260 and 32070946), the Natural Science Foundation of Shanghai (number 16ZR1429500), the Shanghai Pujiang Program (number 16PJ1408700), the Shanghai “Rising Stars of Medical Talent” Youth Development Program, the Outstanding Youth Medical Talents [SHWJRS (2021)-99], and the Fundamental Research Funds for the Central Universities (HUST: 2015ZHYX007 and 2018KFYYXJJ076).

## Conflict of Interest

The authors declare that the research was conducted in the absence of any commercial or financial relationships that could be construed as a potential conflict of interest.

## Publisher’s Note

All claims expressed in this article are solely those of the authors and do not necessarily represent those of their affiliated organizations, or those of the publisher, the editors and the reviewers. Any product that may be evaluated in this article, or claim that may be made by its manufacturer, is not guaranteed or endorsed by the publisher.
