# Supplementary material for: Cryptococcus neoformans Infection Induces IL-17 Production by Promoting STAT3 Phosphorylation in CD4+ T Cells
Source: Front Immunol. 2022 May 27;13:872286. doi: 10.3389/fimmu.2022.872286 (PMC9197778; doi:10.3389/fimmu.2022.872286)
Supplement: Supplementary file 1 [file DataSheet_1.pdf]

Figure S1

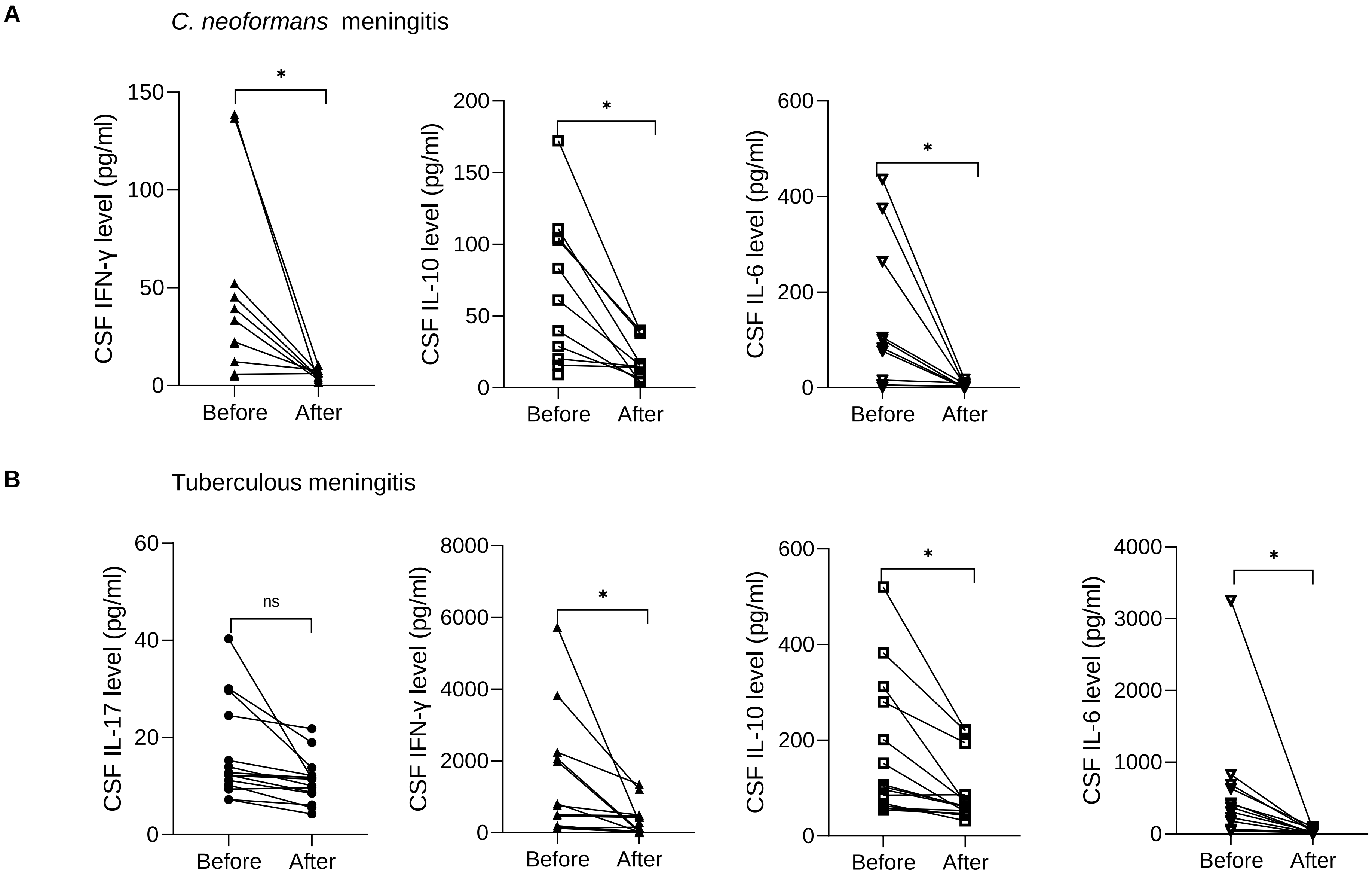

Figure S1: Pathogen-specific therapies minimizes cytokine levels in cerebrospinal fluid of patients with meningitis. Patients with *C. neoformans* meningitis or Tuberculous meningitis were treated with standard pathogen-specific therapies (anti-fungal therapy or anti-tuberculous therapy), cytokine levels in cerebrospinal fluid before and after anti-fungal therapy (A) or anti-tuberculous therapy (B) were evaluated by ELISA. *$0.01<p<0.05$.

Figure S2

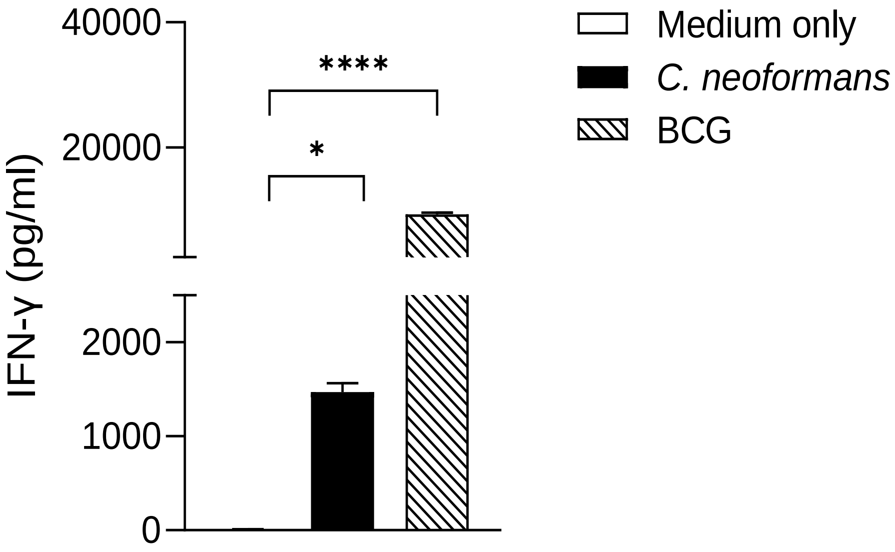

Figure S2: *C. neoformans* and BCG differentially stimulate IFN-γ expression by PBMCs. PBMCs were stimulated with or without *C. neoformans* or BCG for 3 days, supernatant IFN-γ was detected by ELISA, data was shown as mean ± SE of three individual experiments. *0.01<p<0.05,****p < 0.0001.

Figure S3

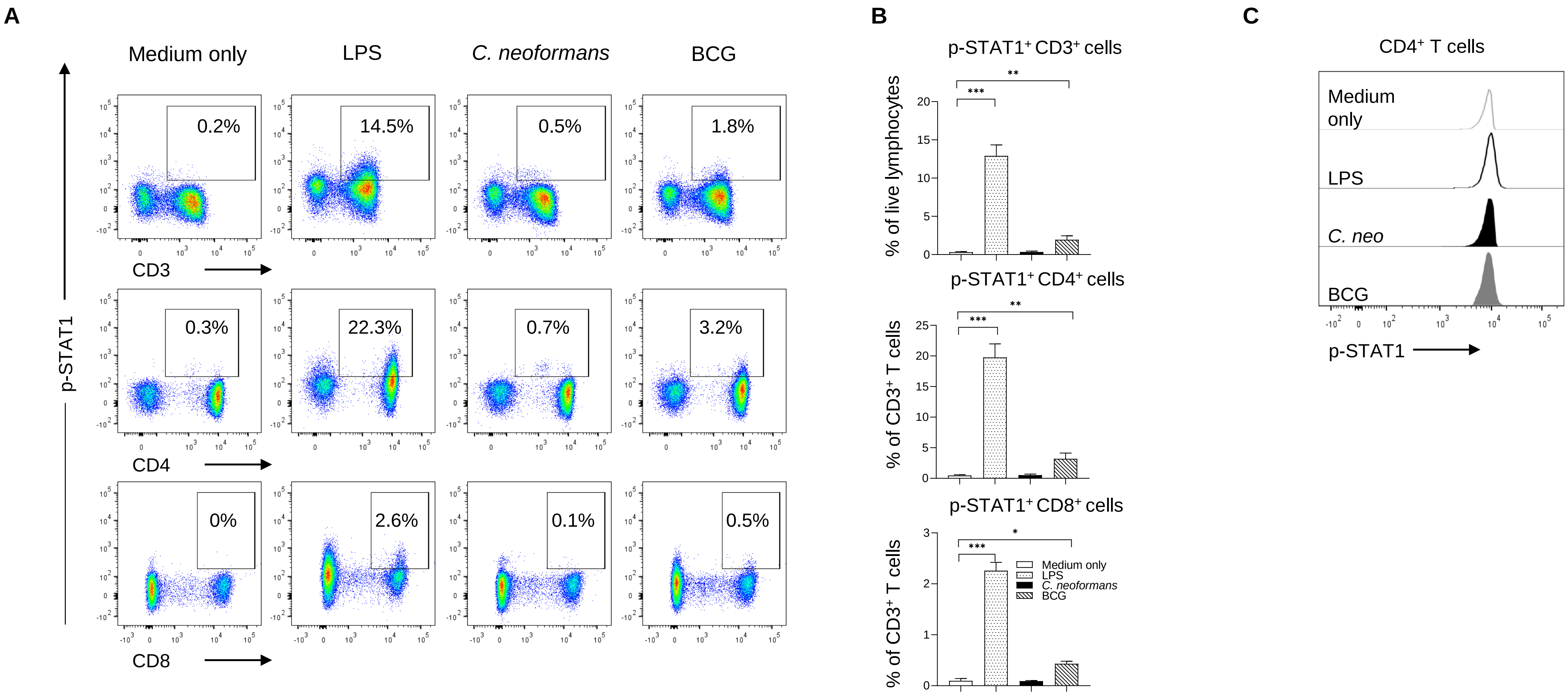

Figure S3: *C. neoformans* stimulates phosphorylation of STAT1 in CD4$^+$ T cells. PBMCs were stimulated for 30 minutes with LPS , *C. neoformans*, or BCG, phosphorylation of STAT1 in T cell subsets (A and B) and its fluorescence intensity was analyzed by flow cytometry (C). *$0.01<p<0.05$, **$0.001<p<0.01$, ***$<0.0001<p<0.001$.

Figure S4

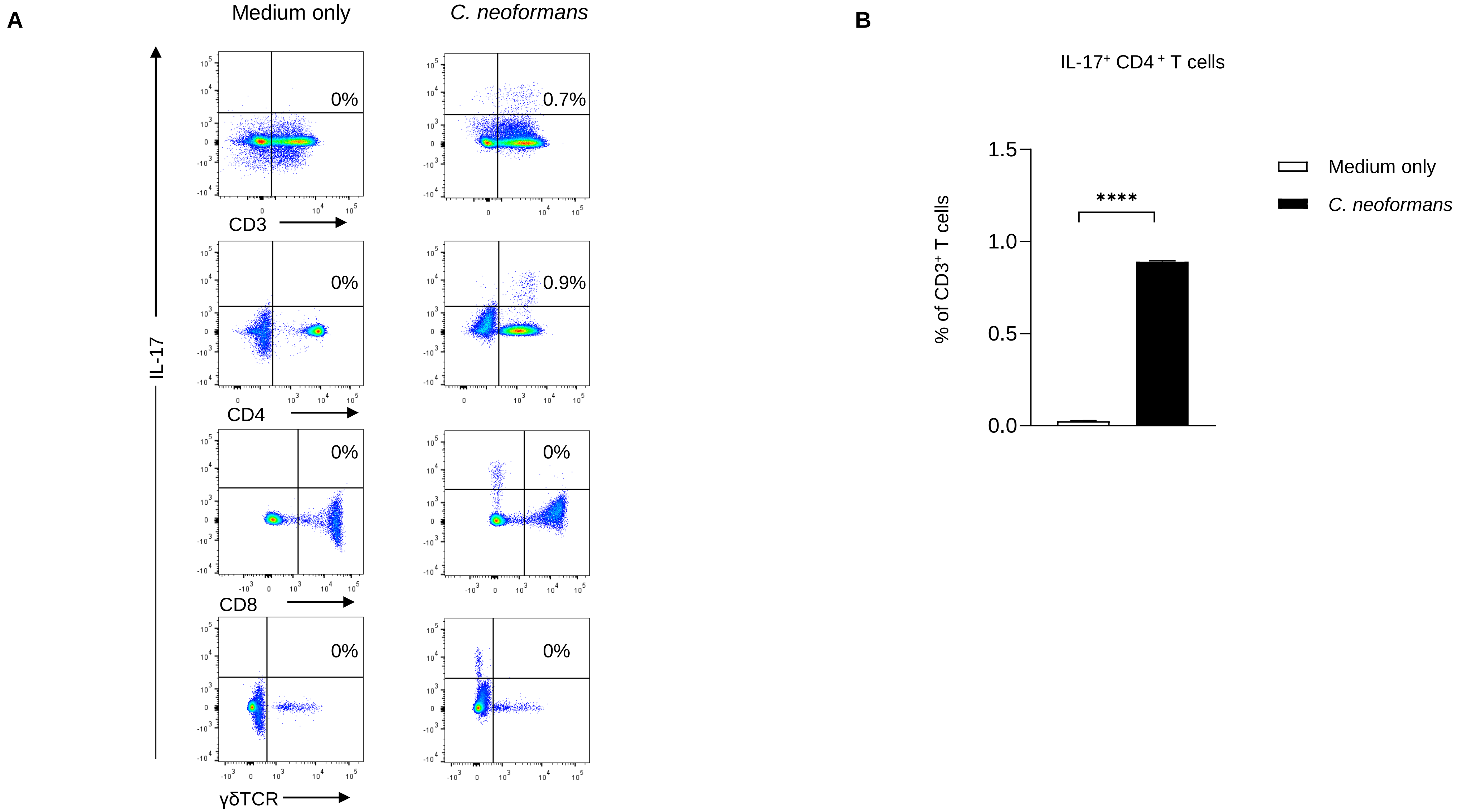

Figure S4: *C. neoformans* stimulates IL-17 production by CD4+ T cells *in vitro.* PBMCs were stimulated with *C. neoformans* for 30min plus protein transport inhibitor cocktail for 5 hours before collected cells, IL-17-producing cells were analyzed by flow cytometry: representative data of IL-17+ T cells in αβTCR+ T cells(CD3+γδTCR- cells) and γδTCR+ T cells (A), and percentages of IL-17+ CD4+ T cells in live CD3+ cells (B). **** $p < 0.0001$.
